# Effectiveness and safety of intense pulsed light therapy for dry eye symptoms due to meibomian gland dysfunction—A systematic review and meta‐analysis

**DOI:** 10.1111/aos.16802

**Published:** 2024-11-29

**Authors:** Nathalie Peira, Elham Mohammed Ali, Naama Kenan Modén, Eva Fjellgren, Claes Lennmarken, Monica Hultcrantz

**Affiliations:** ^1^ HTA Region Stockholm, Center for Health Economics, Informatics and Health Care Research (CHIS) Stockholm Health Care Services Stockholm Sweden; ^2^ Department of Learning, Informatics, Management and Ethics Karolinska Institutet Stockholm Sweden

**Keywords:** dry eye disease, dry eyes, intense pulsed light therapy, meibomian gland dysfunction, systematic review

## Abstract

**Background:**

Dry eye disease is a common ocular condition, affecting quality of life and function for many people worldwide. This systematic review evaluates the effectiveness and safety of intense pulsed light (IPL) therapy for dry eye symptoms due to meibomian gland dysfunction (MGD), as they are not yet established.

**Methods:**

Comprehensive searches were conducted in January 2024 (Cochrane Library, Embase, MEDLINE). We included randomized controlled studies assessing the effects of IPL on dry eye symptoms or any adverse effects in patients with MGD. Two authors independently screened studies and evaluated the risk of bias. Meta‐analysis (random effects model) and Grading of Recommendations Assessment, Development and Evaluation (GRADE) were used to synthesize results and assess certainty of evidence.

**Results:**

In total, 13 studies were included in the review. The risk of bias was assessed as moderate in most results primarily due to the risk of selective reporting. The synthesis and GRADE assessment show that IPL probably results in a clinically relevant reduction in symptoms of dry eyes when compared to placebo (MD = −16 OSDI points; 95% CI = −20 to −12). However, whether the effect is clinically relevant when used as an add‐on to standard care is still unclear (MD = −7 OSDI points; 95% CI = −12 to −3). Furthermore, the evidence on adverse events is very uncertain.

**Conclusion:**

Although IPL probably results in a clinically relevant reduction in symptoms of dry eyes when compared to placebo, in practice IPL would typically be used as an add‐on to standard treatment. It is therefore essential with further studies investigating whether this use would give a clinically relevant reduction in symptoms, and what the potential harm of IPL is when treating MGD.

## 1 BACKGROUND

Dry eye disease (DED) is a common ocular condition affecting approximately 350 million individuals globally. It has two primary classifications, aqueous‐deficient dry eye and evaporative dry eye. Aqueous‐deficient dry eye occurs when the lacrimal glands fail to produce enough aqueous tears (Stapleton et al., 2017). Evaporative dry eye results from increased tear evaporation caused by factors such as meibomian gland dysfunction (MGD) (Stapleton et al., 2017). Around 10%–15% of patients with DED experience aqueous‐deficient dry eye alone, with the vast majority, over 85%, having an evaporative component (Sheppard & Nichols, 2023).

Meibomian gland dysfunction has been identified as the primary cause of evaporative DED (Sheppard & Nichols, 2023). Meibomian glands secrete meibum which is an oily substance that forms the lipid layer of the tear film (Nichols et al., 2011). Meibomian gland dysfunction involves decreased meibum secretion or altered meibum composition, leading to disruption of the tear film lipid layer and accelerated tear evaporation (Mishra et al., 2023).

Patients with MGD experience symptoms like burning, itching and grittiness which interfere with daily activities, and visual issues such as blurred vision impede tasks requiring clear sight (Lemp et al., 2012). These persistent symptoms often lead to anxiety and depression, with affected individuals reporting higher emotional distress (Messmer, 2015). Current treatment modalities for MGD primarily focus on alleviating symptoms and improving meibomian gland function. Conventional approaches include warm compress therapy, eyelid hygiene, lubricating eye drops and oral supplements such as omega‐3 fatty acids (Blackie et al., 2015). The existing treatment has varying degrees of effectiveness, with many patients experiencing only partial or temporary relief of symptoms (Liu et al., 2022).

Intense pulsed light (IPL) therapy has emerged as a possible intervention for the treatment of MGD, although originally developed for cosmetic dermatology (Rong et al., 2018a). Intense pulsed light therapy involves the use of a high‐output flash lamp emitting a broad spectrum of non‐coherent light typically ranging from 500 nm to 1200 nm. Intense pulsed light therapy is typically administered in multiple sessions over several months (Cote et al., 2020). Two commonly used devices are the M22 Optima device (Lumenis Ltd., US) and the E‐eye device (E‐Swin, France) (Cote et al., 2020). Intense pulsed light therapy is generally unsuitable for individuals with darker skin tones (types V to VI) due to the risk of hypopigmentation and scarring. Other contraindications include certain medical conditions and medications that increase photosensitivity, necessitating precautions such as the use of eye shields to prevent eye injury from IPL wavelengths (Cote et al., 2020).

A Cochrane review published in 2020 underscored the scarcity of randomized controlled trial (RCT) evidence on IPL's efficacy and safety for MGD (Cote et al., 2020; Sullivan et al., 2014). The Cochrane review noted that there were several ongoing studies, many of which have now been published. Although we have identified several reviews on the topic published after the Cochrane review, we have assessed these as having high risk of bias mainly due to a lack of transparent and reproducible search strategies. Thus, it is now timely to update the Cochrane review and provide an up‐to‐date understanding of IPL's effectiveness and safety.

In this systematic review, we assess the effects on symptoms of dry eyes considering the minimal important difference (MID), which ensures that potential improvements are not just statistically significant but also clinically relevant (Sheppard & Nichols, 2023) and can be weighed against potential harm.

## 2 METHODS

### 2.1 Study design

This systematic review follows the methodology reported in the Cochrane Handbook (Higgins et al., 2022) and is reported according to the PRISMA 2020 reporting system (Page et al., 2021). The protocol for this review was prospectively registered in PROSPERO (CRD42023457420). The review was conducted as part of a project at HTA Region Stockholm, and the report will be published in Swedish on the organization's webpage (https://www.chis.regionstockholm.se/hta/).

### 2.2 Eligibility criteria

The specific eligibility criteria were:

- Population: adults (18 years or older) diagnosed with MGD or evaporative DED.
- Intervention: IPL therapy for treating MGD or evaporative DED
- Comparison: standard treatment (e.g. warm compresses, or artificial tears), placebo treatment (e.g. sham IPL) or no treatment. We have included studies with meibomian gland expression as a co‐intervention if performed in both intervention and control groups.
- Outcomes: dry eye symptoms measured by validated scales such as Ocular Surface Disease Index (OSDI), the Standard Patient Evaluation of Eye Dryness (SPEED), or visual analogue Scale (VAS) and evaluation of any adverse events reported in the studies.
- Study Design: Randomized controlled trials.

### 2.3 Information source and search strategy

The search was an update of the search performed in the Cochrane systematic review published in 2020 (Cote et al., 2020). Initial comprehensive searches were conducted on the 27 to 30 of June 2023 in Cochrane Library, Embase (Elsevier) and MEDLINE (Ovid). The search was updated on 17–18 January 2024. For the full search strategy see Data S1.

### 2.4 Selection process

The selection of studies was made from the studies included in the review conducted by Cochrane and from the searches conducted by HTA Region Stockholm. Two authors independently reviewed the titles and abstracts of the studies identified. Articles whose abstracts met the selection criteria, or where there was uncertainty as to whether they met the criteria, were viewed in full text. The full texts were reviewed independently by two authors. Any disagreements were resolved through discussion until consensus was reached. Full texts that did not meet the selection criteria were excluded and the reason for exclusion was noted (see Data S2).

### 2.5 Data collection process and data items

Data were extracted by one author following a predefined structure and verified by another author. Data extracted were: publication information; study design; setting; recruitment; study population (number of participants; characteristics at baseline); inclusion criteria; follow‐up; details of the intervention and comparison; drop‐outs; outcome (dry eye symptoms at 3 months follow‐up); and reported adverse events.

### 2.6 Study risk of bias assessment

Risk of bias was assessed by two authors independently using the ROB 2‐tool (Sterne et al., 2019). Any disagreements were resolved through discussion until consensus was reached.

### 2.7 Effect measures

If dry eye symptoms were measured with more than one scale, we had the following priority list: (1) OSDI, (2) SPEED, (3) VAS. We judged that OSDI and SPEED measure the same underlying construct of dry eye symptoms and as such were similar enough to be combined in the same meta‐analysis (Guyatt et al., 2013). SPEED has been validated against OSDI and found to behave similarly (Hashmani et al., 2021; Ngo et al., 2013). To combine the questionnaires, we converted SPEED scores according to the method recommended by GRADE (Grading of Recommendations Assessment, Development and Evaluation; (Guyatt et al., 2013)). In short, the conversion is done by calculating the effect size for each study in standard mean difference (SMD) and multiplying by a standard deviation common for the instrument of interest. For this, we used the pooled standard deviation of OSDI for all control groups at baseline of the included studies.

### 2.8 Synthesis methods

Results of the included studies were synthesized in a meta‐analysis using a random effect model estimating mean effect size with 95% confidence intervals (CI) and illustrated with forest plots (all done in the software RevMan). Pre‐planned subgroup analyses were: type of IPL devices used, severity of symptoms and treatment length.

### 2.9 Reporting bias assessment

Potential publication bias was assessed using a funnel plot (from the software RevMan) using results from all included studies. A search for published protocols (April 2024 in ClinicalTrials.gov) was also conducted to estimate the number of studies that had been registered but not yet published.

### 2.10 Certainty assessment

The certainty of evidence was assessed using GRADE (Schünemann et al., 2013). The certainty of evidence represents the certainty that the true effect is above or below a specified threshold (Hultcrantz et al., 2017; Zeng et al., 2021). In this review, we used a defined minimal important difference (MID) for the certainty assessments. The MID was selected for the OSDI scale based on a review of MIDs showing that they could range between 7 and 9.9 across severity levels. We used the lower end of this range, an MID of 7, since the effect size of a meta‐analysis represents a weighted average across study participants which means that approximately half of the participants have a larger effect than the average. Using GRADE, the certainty of evidence for each outcome was assessed as high, moderate, low or very low. The assessment is based on five domains; risk of bias, inconsistency, imprecision, indirectness and publication bias.

## 3 RESULTS

### 3.1 Study selection

**FIGURE 1:**
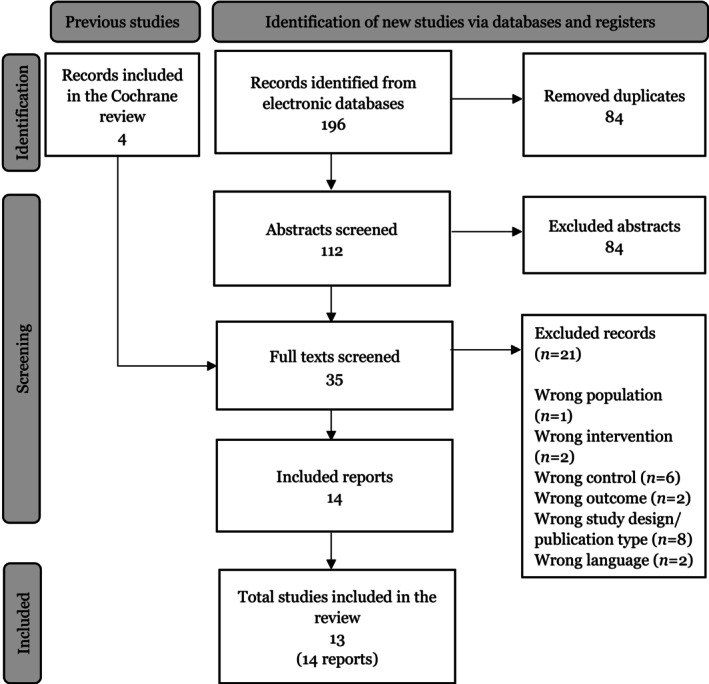
Flow diagram of identification, screening and included studies.

From the records identified through searches in the electronic databases, 112 unique titles and abstracts were independently reviewed by the authors for potential inclusion. Out of these, 35 reports were deemed potentially eligible and proceeded to full‐text screening. Among these, 21 were excluded primarily due to wrong study design and inappropriate control groups (excluded studies with reasons for exclusion are listed in Data S2). The four reports included in the Cochrane review were screened in full text and were all included in the review. In total, 13 studies (14 articles) were included in the review (for more details see Figure 1) (Arita et al., 2019; Chen et al., 2021, 2023; Craig et al., 2015; Li et al., 2023; Piyacomn et al., 2020; Qin et al., 2023; Rong et al., 2018a, 2018b; Song et al., 2022; Toyos et al., 2022; Xue et al., 2020; Yan et al., 2021; Zarei‐Ghanavati et al., 2022).

### 3.2 Study characteristics

The participants in the included studies had severe evaporative disease or meibomian gland dysfunction with mostly severe symptoms of dry eyes. Intense pulsed light therapy was performed over two to four sessions in all but one study. Eight studies used the IPL system called M22 and three used E‐eye. All studies reported data for dry eye symptoms. Most commonly (9/13) the OSDI rating scale was used (min = 0, max = 100; higher = worse). The other studies used the SPEED rating scale (min = 0, max = 28, higher = worse). Two studies were excluded from the meta‐analysis (Craig et al., 2015; Rong et al., 2018a, 2018b) because they compared different eyes from the same patient with each other and did not report effect sizes. In this review, we have focused on two clinical scenarios; (1) receiving IPL as compared to no treatment at all (2) receiving IPL as an add‐on to standard. For the effects on symptoms of dry eyes, the results from these two scenarios are presented separately below. All included studies are described in more detail in Data S3.

#### 3.2.1 IPL compared to no treatment

The studies comparing IPL with no treatment or sham (placebo) treatment are included in this comparison. IPL was compared to sham (placebo) in 3 RCTs (Chen et al., 2023; Qin et al., 2023; Xue et al., 2020) and to no treatment in one study (Li et al., 2023). In total, the meta‐analysis included 280 participants; 157 received IPL and 123 no treatment. Participants were all adults (mean age between 28 and 53) and had severe evaporative disease (Chen et al., 2023;Li et al., 2023; Qin et al., 2023) or meibomian gland dysfunction (Xue et al., 2020) with mostly severe symptoms of dry eyes (Chen et al., 2023; Qin et al., 2023). In one study, inclusion criteria allowed participants to have mild‐to‐severe symptoms, but mean baseline symptom scores indicate that the included participants had severe symptoms (Li et al., 2023). In another study, the severity was not reported (Xue et al., 2020). Intense pulsed light therapy was performed over two (J. Chen et al., 2023), three (Li et al., 2023; Qin et al., 2023), or four sessions (Xue et al., 2020). Three studies used the IPL system called M22 (Chen et al., 2023; Li et al., 2023; Qin et al., 2023), and one study used E‐eye (Xue et al., 2020).

#### 3.2.2 IPL as an add‐on compared to standard treatment

The studies comparing IPL as an add‐on to standard treatment with standard treatment alone are included in this comparison. The comparison was done in seven RCTs (Arita et al., 2019; Chen et al., 2021; Piyacomn et al., 2020; Song et al., 2022; Toyos et al., 2022; Yan et al., 2021; Zarei‐Ghanavati et al., 2022). In total, the meta‐analysis included 595 adult participants (mean age between 28 and 61); 306 received IPL as an add‐on and 289 standard treatment alone. These participants all had severe meibomian gland dysfunction, with most experiencing severe dry eye symptoms as indicated by baseline OSDI ratings (Chen et al., 2021; Piyacomn et al., 2020; Song et al., 2022; Toyos et al., 2022; Zarei‐Ghanavati et al., 2022). In two studies, it is less straightforward to estimate symptom severity because they were measured with SPEED and are not reported at baseline (Arita et al., 2019; Yan et al., 2021). Intense pulsed light therapy was performed over three to four sessions in all but one study that used eight sessions (Arita et al., 2019). Five studies used the IPL system called M22 (Arita et al., 2019; Chen et al., 2021; Song et al., 2022; Toyos et al., 2022; Yan et al., 2021), and two studies used E‐eye (Piyacomn et al., 2020; Zarei‐Ghanavati et al., 2022).

### 3.3 Risk of bias in studies

**FIGURE 2:**
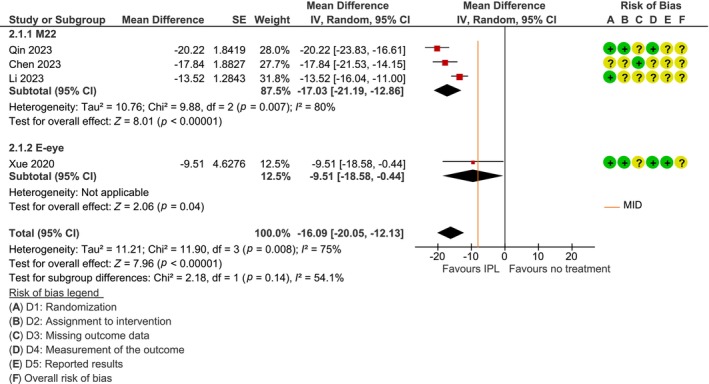
Forest plot for the effect of IPL on dry eye symptoms using OSDI (min = 0, max = 100; higher = worse) compared to no treatment.

**FIGURE 3:**
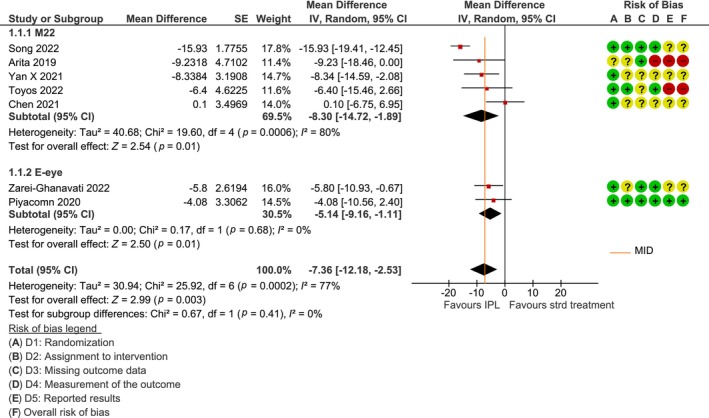
Forest plot for the effect of IPL as an add‐on on dry eye symptoms using OSDI (min = 0, max = 100; higher = worse) compared to standard treatment alone.

The risk of bias was assessed as moderate in most studies with the most consistent risk being that of selective reporting (see Figures 2 and 3). Additionally, most studies did not have a pre‐registered protocol.

#### 3.3.1 IPL compared to no treatment

The risk of bias was assessed as moderate in all studies. In two studies, both participants and personnel were blinded to group assignment (Qin et al., 2023; Xue et al., 2020), and two studies were unblinded (Chen et al., 2023; Li et al., 2023). The lack of pre‐registered protocols was considered the greatest risk of bias. One study had a pre‐registered protocol, but the symptom score outcome was not described in the protocol (Xue et al., 2020).

#### 3.3.2 IPL as an add‐on compared to standard treatment

The risk of bias was assessed as moderate in five studies (Chen et al., 2021; Piyacomn et al., 2020; Song et al., 2022; Yan et al., 2021; Zarei‐Ghanavati et al., 2022) and high in two (Arita et al., 2019; Toyos et al., 2022). In three studies, both participants and testers were blinded to group assignment (Piyacomn et al., 2020; Song et al., 2022; Toyos et al., 2022), and four studies were unblinded (Arita et al., 2019; Chen et al., 2021; Yan et al., 2021; Zarei‐Ghanavati et al., 2022). All but one study (Piyacomn et al., 2020) lacked pre‐registered protocols. This was considered to be the greatest risk of bias.

### 3.4 Results of individual studies and synthesis

The results from each individual included study are presented in Data S3. In the following, we present the results from our analysis per outcome, starting with the effect on dry eye symptoms for the two comparators and then addressing adverse effects.

#### 3.4.1 Effect on dry eye symptoms

##### IPL compared to no treatment

All four studies have reported data for dry eye symptoms and have used the same rating scale (OSDI; min = 0, max = 100; higher = worse). The outcome is reported as the number of points on the scale. At baseline, patients rated their symptoms between 28 and 42 points on average. According to the severity categories of OSDI, this means that the participants as a group had moderate (23–32 points) to severe (33–100 points) symptoms.

Treatment with IPL shows a reduction in symptoms of dry eyes by an average of −16 points (95% CI = −20 to −12 points), compared to no treatment (see forest plot in Figure 2). For the assessment of the certainty of evidence, see Table 2.

No subgroup analysis was performed on severity or treatment duration, as there would have been few studies in the groups, and the studies were considered relatively similar (moderate‐to‐severe severity and 2 to 4 IPL sessions).

The subgroup analysis for the type of IPL machine (M22/E‐eye) was performed, even though only one study used the E‐eye. This was done because we could not assess whether the different machines provide sufficiently similar effects to be combined. The subgroup analysis did not show any statistically significant difference (*p* = 0.14; see also Figure 2). Since no credible effect modifiers were identified, we used the combined estimate.

##### IPL as an add‐on compared to standard treatment

All seven studies that are included in the meta‐analysis have reported data for dry eye symptoms. Five of those (Chen et al., 2021; Piyacomn et al., 2020; Song et al., 2022; Toyos et al., 2022; Zarei‐Ghanavati et al., 2022) have used OSDI as rating scale (min = 0, max = 100; higher = worse) and two (Arita et al., 2019; Yan et al., 2021) have used SPEED (min = 0, max = 28, higher = worse). The outcome is reported as the number of points on OSDI (data from SPEED have been converted to OSDI, for details see methods section). At baseline, patients rated their symptoms between 23 and 60 points on average. According to the severity categories of OSDI, this means that the participants as a group had moderate‐ (23–32 points) to‐severe (33–100 points) symptoms.

Treatment with IPL shows a reduction in symptoms of dry eyes by an average of 7 points (95% CI = −12 to −3 points), compared to standard treatment alone (see forest plot in Figure 3). For the assessment of the certainty of evidence, see Table 2.

No subgroup analysis was performed on severity or treatment duration, as there were few studies in each subgroup, and the studies were considered relatively similar (moderate‐to‐severe severity and three to four IPL sessions). The subgroup analysis for the type of IPL machine (M22/E‐eye) was performed, even though only two studies used the E‐eye (Piyacomn et al., 2020; Zarei‐Ghanavati et al., 2022). We did this because we could not assess whether the different machines provide sufficiently similar effects to be combined. The subgroup analysis did not show any statistically significant difference (*p* = 0.41; see also Figure 3). Since no effect modification was identified, also for this comparator we used the combined estimate.

One of the studies (Arita et al., 2019) has used data from both eyes for each patient, and it is unclear whether the analyses were done per eye or per patient. Based on a sensitivity analysis, we chose to include the study despite this uncertainty, as the results were not affected (result with the study: MD = −7 (95% CI = −12 to −3); result without the study: MD = −7 (95% CI = −12 to −2)).

#### 3.4.2 Adverse effects of IPL treatment

Of the 13 included studies, 10 measured adverse events. These are included in this analysis, regardless of whether IPL was used alone or as an add‐on to standard treatment (Arita et al., 2019; Chen et al., 2021, 2023; Piyacomn et al., 2020; Qin et al., 2023; Rong et al., 2018a, 2018b; Song et al., 2022; Xue et al., 2020; Yan et al., 2021; Zarei‐Ghanavati et al., 2022). Adverse events were reported sporadically and without any possibility of quantitative comparison between intervention and comparison groups. As such, no meta‐analysis was possible.

Of the 10 studies that measured adverse events, four studies reported adverse events and six studies reported no adverse events. Overall, the reported adverse events were mild and transient (see Table 1).

**TABLE 1:** Reported adverse events.

Study (reference), No. participants	Adverse events	Comment
Chen 2021 (Chen et al., 2021) *n* = 67	Mild pain and burning sensation during the IPL therapy, but no sustained skin injury occurred after the treatment. Overall, no complications were observed	Mild and transient adverse events
Rong 2018 (Rong et al., 2018a) *n* = 44	Mild pain and burning during IPL treatment in five patients, mild redness of the eyelids in their study eyes and one patient suffered partial eyelash loss. No irreversible eyelid skin injury occurred, and no intraocular inflammation, iris transillumination defects, or ocular surface or fundus injuries were observed	Mild and transient adverse events
Yan 2021 (Yan et al., 2021) *n* = 120	No adverse events related to the device or the procedure. In the control group, one patient developed lower eyelid oedema during the study and had to stop participation in the study	No adverse events in the intervention group
Chen 2023 (Chen et al., 2023) *n* = 44	No systemic adverse events were observed during the study. After treatment, in rare cases eye irritation, conjunctival hyperaemia, eye pain and the skin around the eye becoming sensitive and fragile may occur. No special treatment was required for these to relieve and subside within a few hours	Mild and transient adverse events

### 3.5 Reporting biases

Financial interests in the area makes the risk of reporting bias higher, including outcome reporting bias which was considered in the risk of bias assessments (see Figures 2 and 3), and publication bias which was assessed by a funnel plot. The studies included in the funnel plot were similarly small and did not exhibit the typical inverted funnel pattern, the results were inconclusive (see Data S4). In addition, we attempted to identify potential studies that were not published due to unfavourable results by searching for protocols without corresponding completed studies. However, our findings in this regard were limited.

### 3.6 Certainty of evidence

The assessments of certainty of evidence for each outcome is presented in Table 2.

**TABLE 2:** Treatment with IPL, summary of effect and certainty of evidence.

Outcome comparison	Effect (95% CI) MID	No. participants (No. studies)	Certainty of the evidence reasons for rating down	Summary statement
Symptoms of dry eyes^f^ IPL versus no treatment	MD = −16 points (−20 to −12) MID = 7 points	280 (4 RCT)	⊕⊕⊕⊖ Moderate Risk of bias‐1^a^	IPL probably results in a clinically relevant reduction in symptoms compared to no treatment at all
Symptoms of dry eyes^f^ IPL as add‐on versus standard treatment alone	MD = −7 points (−12 to −3) MID = 7 points	590 (7 RCT)	⊕⊖⊖⊖ Very low Risk of bias‐1^a^ Inconsistency‐1^b^ Precision‐1^c^	It is very uncertain whether IPL results in a clinically relevant reduction in symptoms compared to standard treatment
Adverse effects of IPL for treating MGD or evaporative DED	4/10 studies report mild and transient adverse events	746 (10 RCT)	⊕⊖⊖⊖ Very low Risk of bias‐2^d^ Precision‐2^e^	The evidence is very uncertain about the adverse effects of IPL

*Note*: Population: Adults (≥18 years) with dry eye symptoms due to meibomian gland dysfunction or evaporative dry eye disease.

Abbreviations: CI, confidence interval; MD, mean difference; MID, minimal important difference; RCT, randomized controlled study; SMD, Standardized mean difference.

^a^ Concerns due to risk of bias, mainly selective reporting, together with some concerns for potential publication bias since pre‐registered protocols are rare in this research area.

^b^ Concerns for inconsistency as nearly half of the studies' point estimates indicate clinically non‐relevant effects.

^c^ Concerns for imprecision as the CI of the pooled estimate includes a clinically non‐relevant effect.

^d^ Serious concerns due to risk of outcome reporting bias as the outcome was sporadically reported together with the fact that pre‐registered protocols are rare in this research area.

^e^ Serious concerns for imprecision due to scarce data and no reporting of number of events leading to a non‐estimable effect size.

^f^ OSDI 0–100, higher = worse.

## 4 DISCUSSION

This review expands on the 2020 Cochrane review (Cote et al., 2020), which highlighted the scarcity of evidence from RCTs on IPL's efficacy and safety for MGD, necessitating further studies to inform evidence‐based clinical decision making. Unlike the Cochrane review, which included only three studies, this updated systematic review incorporates 13 studies enabling comparisons on two clinical scenarios; IPL as compared to no treatment and IPL as an add‐on to standard treatment.

### 4.1 Strengths and limitations

This review focused on symptoms of dry eyes, which is a patient's important outcome, and presents the results using the most common scale to support interpretability. The effects of IPL on dry eye symptoms were assessed considering a threshold for a MID. This ensured that the demonstrated improvements are not just statistically significant but also assessed in relation to what is considered a clinically relevant effect. Limitations considered were that the number of studies and participants were relatively small. Furthermore, subgroup analysis was inconclusive due to the disproportionate number of RCTs included for comparison of different types of IPL devices. This review is limited to MGD and dry eye symptoms. There are other conditions such as patients with severe aqueous deficient dry eye and MGD or ocular rosacea that might be relevant for investigations of treatment effects of IPL. However, these fall outside the scope of this particular review.

## 5 CONCLUSIONS AND IMPLICATIONS FOR RESEARCH AND PRACTICE

Intense pulsed light therapy probably results in a clinically relevant reduction in dry eye symptoms associated with MGD when compared to no treatment. The certainty of evidence was moderate, primarily due to the risk of selective reporting. However, while IPL therapy as an add‐on showed a reduction in dry eye symptoms compared to standard treatments, it is still unclear whether the effect is clinically relevant. The certainty of evidence was very low due to risk of bias, inconsistency in results and imprecision. The evidence is also very uncertain regarding adverse effects of IPL.

To reduce the risk of selective reporting and publication bias, future studies should have pre‐registered protocols. The lack of pre‐registered protocols is noteworthy in this area, given the financial interests behind the IPL devices and private clinics offering these treatments. Furthermore, new studies should investigate IPL specifically as an add‐on to standard treatment, since this is how IPL would typically be used in clinical practice. At this point, there are no robust data supporting a benefit for patients adding IPL to already existing treatments.

## FUNDING INFORMATION

This systematic review was conducted at HTA Region Stockholm.

## CONFLICT OF INTEREST STATEMENT

The authors declare no conflicts of interest.

## ETHICS STATEMENT

Not applicable.

## SYSTEMATIC REVIEW REGISTRATION

PROSPERO CRD42023457420.

## Supporting information


Data S1.

Data S2.

Data S3.

Data S4.

## Data Availability

All data generated or analysed during this study are included in this published article and its supplementary information files.
