# Supplementary material for: Effectiveness and safety of intense pulsed light therapy for dry eye symptoms due to meibomian gland dysfunction—A systematic review and meta‐analysis
Source: Acta Ophthalmol. 2024 Nov 29;103(4):371–9. doi: 10.1111/aos.16802 (PMC12069959; doi:10.1111/aos.16802)
Supplement: Supplementary file 1 — Data S1. [file AOS-103-371-s002.docx]

## Supporting Information S1 –Search strategies

**Search:** Updated search from Cote et al. 2020 “Intense pulsed light (IPL) therapy for the treatment of meibomian gland dysfunction” (Cochrane). Their search was done in August 2019. No limitations on language and publication date.

| Cochrane Library via Wiley 2023-06-27  (Search updated 2024-01-17, see below) | | |
| --- | --- | --- |
|  | **Search terms** | **Items found** |
|  | **Patient, problem** |  |
|  | MeSH descriptor: [Dry Eye Syndromes] explode all trees | 1 759 |
|  | MeSH descriptor: [Tears] explode all trees | 783 |
|  | MeSH descriptor: [Meibomian Glands] explode all trees | 130 |
|  | MeSH descriptor: [Eyelids] explode all trees | 1 274 |
|  | MeSH descriptor: [Blepharitis] explode all trees | 160 |
|  | MeSH descriptor: [Keratoconjunctivitis] explode all trees | 730 |
|  | (meibomian):ti,ab,kw | 648 |
|  | (dry NEXT eye*):ti,ab,kw | 3 598 |
|  | ((eye NEXT lid*) OR eyelid*):ti,ab,kw | 2 491 |
|  | (tear NEXT film):ti,ab,kw | 1 576 |
|  | (tear NEXT stabil*):ti,ab,kw | 82 |
|  | (tear NEXT instab*):ti,ab,kw | 12 |
|  | (“evaporative dry” NEXT eye*):ti,ab,kw | 87 |
|  | (meibum):ti,ab,kw | 122 |
|  | (lipid*):ti,ab,kw | 51 169 |
|  | (“eye dryness”):ti,ab,kw | 166 |
|  | (MGD):ti,ab,kw | 10 607 |
|  | **1 OR 2 OR 3 OR 4 OR 5 OR 6 OR 7 OR 8 OR 9 OR 10 OR 11 OR 12 OR 13 OR 14 OR 15 OR 16 OR 17** | 67 380 |
|  | **Intervention** |  |
|  | MeSH descriptor: [Intense Pulsed Light Therapy] explode all trees | 68 |
|  | MeSH descriptor: [Phototherapy] explode all trees | 4 397 |
|  | (intense near/3 puls*):ti,ab,kw | 795 |
|  | (puls* near/2 light):ti,ab,kw | 477 |
|  | (light near/3 therapy):ti,ab,kw | 3 080 |
|  | (IPL):ti,ab,kw | 446 |
|  | **19 OR 20 OR 21 OR 22 OR 23 OR 24** | 6 392 |
|  | **18 AND 25** | 199 |
| Final | Limits: CENTRAL (Trials), publ. year >= 2019  NOT (ct.gov OR ictrp) | 60 |

[Mesh] = Term from the Medline controlled vocabulary, including terms found below this term in the MeSH hierarchy

:ti,ab,kw= Term found in title, abstract or keywords

* = Truncation

“ “ = Citation Marks; searches for an exact phrase

**search updated:** 2024-01-17, 8 hits

| Embase via Elsevier 2023-06-29  (Search updated 2024-01-18, see below) | | |
| --- | --- | --- |
|  | **Search terms** | **Items found** |
|  | **Patient, problem** |  |
|  | 'keratoconjunctivitis'/exp | 8 738 |
|  | meibomian OR 'meibomian gland'/exp | 4 870 |
|  | dry eye OR 'dry eye'/exp | 52 916 |
|  | eye lid* OR eyelid* OR 'eyelid'/exp | 83 461 |
|  | conjunctiva* OR 'conjunctiva'/exp | 54 714 |
|  | eyelash* OR 'eyelash'/exp | 4 431 |
|  | tear* OR 'lacrimal fluid'/exp | 98 472 |
|  | 'tear film'/exp | 5 552 |
|  | meibomian gland*:ti,ab,kw OR dry eye*:ti,ab,kw OR tear film:ti,ab,kw OR tear stabil*:ti,ab,kw OR tear instab*:ti,ab,kw OR evaporative dry eye*:ti,ab,kw OR meibum:ti,ab,kw OR lipid:ti,ab,kw OR eye dryness:ti,ab,kw OR mgd:ti,ab,kw | 643 367 |
|  | **1 OR 2 OR 3 OR 4 OR 5 OR 6 OR 7 OR 8 OR 9** | 886 879 |
|  | **Intervention** |  |
|  | 'intense pulsed light therapy'/exp | 928 |
|  | (intense NEAR/3 puls*):ti,ab,kw | 2 735 |
|  | (light NEAR/3 therapy):ti,ab,kw | 6 069 |
|  | (puls* NEAR/2 light):ti,ab,kw | 5 749 |
|  | ipl:ti,ab,kw | 5 833 |
|  | **11 OR 12 OR 13 OR 14 OR 15** | 17 395 |
|  | **10 AND 16** | 556 |
|  | ‘crossover procedure’/de OR 'double blind procedure'/de OR 'randomized controlled trial'/de OR 'single blind procedure'/de OR random*:ab,ti OR factorial*:ab,ti OR crossover*:ab,ti OR placebo*:ab,ti OR assign*:ab,ti OR allocat*:ab,ti OR volunteer*:ab,ti OR (cross NEXT/1 over*):ab,ti OR (doubl* NEAR/1 blind*):ab,ti OR (singl* NEAR/1 blind*):ab,ti | 2880 767 |
|  | **17 AND 18** | 109 |
| Final | Limits: Publ. year >= 2019, article, article in press | 53 |

/exp = Includes terms found below this term in the EMTREE hierarchy

/de = Term from the EMTREE controlled vocabulary

:ab,ti,kw= Term found in title and/or abstract and/or keyword

* = Truncation

NEAR/1= one term will be **within** one word of the other in any order

NEXT/1= one term will be within one word of the other in the order in which they're entered

**search updated:** 2024-01-18, hits

| Medline via Ovid 2023-06-30 (2019 to June 2023)  (Search updated 2024-01-18, see below) | | |
| --- | --- | --- |
|  | **Search terms** | **Items found** |
|  | **Patient, problem** |  |
|  | exp Dry Eye Syndromes/ | 4 605 |
|  | exp Eyelids/ | 4 845 |
|  | exp Conjunctiva/ | 2 130 |
|  | exp Eyelashes/ | 174 |
|  | exp Meibomian glands/ | 665 |
|  | exp Blepharitis/ | 192 |
|  | exp Tears/ | 2 180 |
|  | exp Keratoconjunctivitis/ | 295 |
|  | meibomian.tw. OR dry eye*.tw. OR (eyelid* or eye lid*).tw. OR tear film.tw. OR tear stabil*.tw. OR tear instab*.tw. OR evaporative dry eye*.tw. OR meibum.tw. OR lipid*.tw. OR eye dryness.tw. OR MGD.tw. | 108 029 |
|  | **1 OR 2 OR 3 OR 4 OR 5 OR 6 OR 7 OR 8 OR 9** | 113 832 |
|  | **Intervention** |  |
|  | exp Intense Pulsed Light Therapy/ | 105 |
|  | (intense adj3 puls*).tw. | 353 |
|  | (light adj3 therapy).tw. | 966 |
|  | (puls* adj2 light).tw. | 660 |
|  | IPL.tw. | 888 |
|  | **11 OR 12 OR 13 OR 14 OR 15** | 2 296 |
|  | **10 AND 16** | 155 |
|  | randomized controlled trial.pt. OR controlled clinical trial.pt. OR (randomised or randomized).ab,ti. OR placebo.ab. OR drug therapy.fs. OR randomly.ab OR trial.ab OR groups.ab | 1056 661 |
|  | exp animals/ not humans.sh. | 531 964 |
|  | **18 NOT 19** | 939 190 |
| Final | 17 AND 20 | 58 |

.ab. =Abstract

.ti. = Title

.ab,ti. = Abstract or title

exp= Term from the Medline controlled vocabulary, including terms found below this term in the MeSH hierarchy

.fs.= Floating Sub-Heading

.tw = Text word

.pt.= Publication Type

.sh.= Term from the Medline controlled vocabulary

* or $= Truncation (if found at the end of a free text term)

ADJn= positional operator that lets you retrieve records that contain your terms (in any order) within a specified number (n) of words of each other.

**search updated:** 2024-01-18, hits

**total number of hits:** 171 + 25= 196

**after removal of duplicates:** 95 + 17= 112
