# Supplementary material for: Effectiveness and safety of intense pulsed light therapy for dry eye symptoms due to meibomian gland dysfunction—A systematic review and meta‐analysis
Source: Acta Ophthalmol. 2024 Nov 29;103(4):371–9. doi: 10.1111/aos.16802 (PMC12069959; doi:10.1111/aos.16802)
Supplement: Supplementary file 2 — Data S2. [file AOS-103-371-s001.docx]

## Supporting Information S2 – Excluded studies

| Reference | Reason for exclusion |
| --- | --- |
| Chen J, Qin G, Li L, Qi Y, Che H, Huang H, et al. Protocol for a parallel assignment prospective, randomised, comparative trial to evaluate the safety and efficacy of intense pulsed light (IPL) combined with 3% diquafosol (DQS) ophthalmic solution in dry eye syndrome. BMJ Open. 2023;13(8). | Study design/ publication type |
| Chen J, Qin G, Li L, Qi Y, Xia Y, Zhang Q, et al. Correction: the Combined Impact of Intense Pulsed Light Combined and 3% Diquafosol Ophthalmic Solution on Evaporative Dry Eye: a Randomized Control Study (Ophthalmology and Therapy, (2023), 10.1007/s40123-023-00784-z). Ophthalmology and therapy. 2023. | Study design/ publication type |
| Dai PF, Li Y, Tian F, Wang YQ, Ni SS, Wang J. Efficacy comparison of intense pulsed light combined with meibomian gland massage and eyelid fumigation massage in the treatment of MGD-related dry eyes. International Eye Science. 2019;19(12):2101-6. | Language |
| Fukuoka S, Arita R. Comparison of Intense Pulsed Light Therapy on Patients with Meibomian Gland Dysfunction Using AQUA CEL and M22 Devices. Journal of Clinical Medicine. 2022;11(15). | Control |
| Giannaccare G, Pellegrini M, Scalzo GC, Borselli M, Ceravolo D, Scorcia V. Low-Level Light Therapy Versus Intense Pulsed Light for the Treatment of Meibomian Gland Dysfunction: Preliminary Results From a Prospective Randomized Comparative Study. Cornea. 2023;42(2):141-4. | Control |
| Giannaccare G, Rossi C, Borselli M, Carnovale Scalzo G, Scalia G, Pietropaolo R, et al. Outcomes of low-level light therapy before and after cataract surgery for the prophylaxis of postoperative dry eye: A prospective randomised double-masked controlled clinical trial. British Journal of Ophthalmology. 2023. | Intervention |
| Gouws P, Barabas S, Gouws A. Efficacy of Portable 445 nm Laser Versus Intense Pulsed Light Treatment for Dry Eye: A Prospective Randomized Pilot Study. Photobiomodulation, photomedicine, and laser surgery. 2023;41(3):120-4. | Control |
| Huang X, Qin Q, Wang L, Zheng J, Lin L, Jin X. Clinical results of Intraductal Meibomian gland probing combined with intense pulsed light in treating patients with refractory obstructive Meibomian gland dysfunction: a randomized controlled trial. BMC ophthalmology. 2019;19(1):211. | Control |
| Huang Y, Li J, Li DD, Sun JH, Xia X, Peng QH. Meta-analysis of therapeutic effect of intense pulsed light combined with meibomian gland expression on meibomian gland dysfunction related dry eye. International Eye Science. 2023;23(4):616-23. | Study design/ publication type |
| Huo Y, Wan Q, Hou X, Zhang Z, Zhao J, Wu Z, et al. Therapeutic Effect of Intense Pulsed Light in Patients with Sjögren’s Syndrome Related Dry Eye. Journal of Clinical Medicine. 2022;11(5). | Population |
| Jiang X, Yuan H, Zhang M, Lv H, Chou Y, Yang J, et al. The Efficacy and Safety of New-Generation Intense Pulsed Light in the Treatment of Meibomian Gland Dysfunction-Related Dry Eye: A Multicenter, Randomized, Patients-Blind, Parallel-Control, Non-Inferiority Clinical Trial. Ophthalmology and Therapy. 2022;11(5):1895-912. | Control |
| Lei Y, Peng J, Liu J, Zhong J. Intense pulsed light (IPL) therapy for meibomian gland dysfunction (MGD)–related dry eye disease (DED): a systematic review and meta-analysis. Lasers in Medical Science. 2023;38(1). | Study design/ publication type |
| Leng X, Shi M, Liu X, Cui J, Sun H, Lu X. Intense pulsed light for meibomian gland dysfunction: a systematic review and meta-analysis. Graefe's archive for clinical and experimental ophthalmology = Albrecht von Graefes Archiv fur klinische und experimentelle Ophthalmologie. 2021;259(1):1-10. | Study design/ publication type |
| Park Y, Kim H, Kim S, Cho KJ. Effect of low-level light therapy in patients with dry eye: a prospective, randomized, observer-masked trial. Scientific reports. 2022;12(1):3575. | Intervention |
| Piyacomn Y, Kasetsuwan N, Reinprayoon U. Erratum: efficacy and safety of intense pulsed light in patients with meibomian gland dysfunction-a randomized, double-masked, sham-controlled clinical trial (Cornea (2020) 39 (325–332) DOI: 10.1097/ICO.0000000000002204). Cornea. 2020;39(7):E18. | Study design/ publication type |
| Ren X, Chou Y, Wang Y, Chen Y, Liu Z, Li X. Comparison of intense pulsed light and near-infrared light in the treatment of dry eye disease: a prospective randomized study. Acta ophthalmologica. 2021;99(8):e1307‐e14. | Control |
| Xiao Y, Yin HB, Zhang YY, Deng YP. Evaluation of short-term effect of E-eye intense pulsed light combined with meibomian gland expression in the treatment of meibomian gland dysfunction. International Eye Science. 2021;21(1):124-31. | Language |
| Xue AL, Wang MT, Craig JP. Randomised double-masked trial of the cumulative treatment profile of intense regulated pulsed light therapy for meibomian gland dysfunction. Contact lens & anterior eye. 2019;42(6):e28‐e9. | Study design/ publication type |
| Yan S, Wu Y. Efficacy and safety of Intense pulsed light therapy for dry eye caused by meibomian gland dysfunction: a randomised trial. Annals of palliative medicine. 2021;10(7):7857-65. | Outcome |
| Yin XY, Wang H, Zhang ZW, Wang YN, Zhang L, Li MX. Safety and efficacy of intense pulsed light in the treatment of severe chronic ocular graft-versus-host disease. International Eye Science. 2023;23(7):1104-13. | Study design/ publication type |
| Zhang W, Cao X, Yang L, Duan Y, Zhang W. Analysis of Treatment Efficacy of Intense Pulsed Light (M22) for Meibomian Gland Dysfunction with Demodex Mites. Clinical, Cosmetic and Investigational Dermatology. 2023;16:3743-51. | Outcome |
