# Supplementary material for: Effectiveness and safety of intense pulsed light therapy for dry eye symptoms due to meibomian gland dysfunction—A systematic review and meta‐analysis
Source: Acta Ophthalmol. 2024 Nov 29;103(4):371–9. doi: 10.1111/aos.16802 (PMC12069959; doi:10.1111/aos.16802)
Supplement: Supplementary file 3 — Data S3. [file AOS-103-371-s003.docx]

## Supporting Information S3 – Characteristics of included studies

**Notes and abbreviations**

* Calculated by HTA Region Stockholm from values reported in the published article

n= number of participants; NA= not available; RCT= randomized controlled trial; I= intervention group; C= control group; CI= confidence interval; p= probability value; ns= not statistically significant; SD= standard deviation;

| **Author** | **Guanghao Qin** |
| --- | --- |
| **Year** | 2023 |
| **Country** | China |
| **Study design** | RCT |
| **Setting** | He Eye Specialist Hospital, Shenyang |
| **Recruitment** | Participants were recruited between January and October 2021. |
| **Population** | **Gender n (%):** IPL group - 16 females (57.1%); Control group - 11 females (52.4%)  **Mean age (SD)**: IPL group - 28.05 (3.41); Control group - 28.14 (3.53)  **Diagnosis:** severe evaporative DED  **Severity of symptoms**: severe  **Duration of symptoms**: not specified |
| **Inclusion criteria** | Age ≥ 18 years, Fitzpatrick skin types I–IV, capable and willing to comply with treatment and follow-up, diagnosed with severe Dry eye disease  **Exclusion**: Existing ocular trauma, infectious diseases, recent surgical history, skin defects, pigmentation, moles, scars in the treatment area, autoimmune diseases, pregnancy or lactation, Fitzpatrick skin type V or VI |
| **Follow up** | 12 weeks (also 3 weeks and 6 weeks) |
| **Intervention** | Participants received intense pulsed light (IPL) therapy using the M22 IPL system (M22; Lumenis, Yokneam, Israel). The treatment protocol followed the Toyos protocol, involving three sessions separated by 3 weeks, with 12 bilateral light pulses to the periocular and cheek regions. |
| **Participants, n** | IPL group – 30 later ----56 eyes (28 participants); |
| **Drop-outs, n (%)** | IPL group - 2 participants; (6.67%) |
| **Comparison** | sham therapy |
| **Participants, n** | Control group – 30 later---42 eyes (21 participants) |
| **Drop-outs, n (%)** | 9 participants (30%) |
| **Primary outcomes** | **Dry eye symptoms** (OSDI, min= 0; max= 100; higher= worse); mean (SD):  Baseline:  I (n= 28)= 40.98 (7.29)  C (n= 21)= 42.02 (6.57)  Follow up  I (n= 28)= 22.16 (6.08)  C (n= 21)= 42.38 (6.60)  Group difference; mean(95%CI)= NA; p< 0.001 |
| **Reported adverse events** | No adverse effects reported |
| **Comments** | Not clear if the means and standard deviations reported with participant or eye as unit of analysis. Because SD are similar to the studies reporting participants as unit of analysis, data was treated as such. |
| **Risk of bias** | Some concerns |

| **Author** | **Yiqin Chen** |
| --- | --- |
| **Year** | 2021 |
| **Country** | China |
| **Study design** | RCT |
| **Setting** | Eye Hospital and School of Ophthalmology and Optometry, Wenzhou Medical University, Wenzhou, Zhejiang, China |
| **Recruitment** | Patients were enrolled from April to November 2018 at the Eye Hospital of Wenzhou Medical University, Hangzhou. |
| **Population** | **Gender n (%):** Male: 34 (34%), Female: 66 (66%)  **Mean age (SD)**: 46.13 (15.14)  **Diagnosis:** Meibomian gland dysfunction  **Severity of symptoms**: not mentioned  **Duration of symptoms**: not mentioned |
| **Inclusion criteria** | Age ≥ 18 years old, proportion of upper and lower meibomian gland dropout < 50%, the number of upper and lower glands that secrete clear liquid < 6, complete three times of treatment and follow up regularly.  **Exclusion**: Various factors including history of ocular surgery, trauma, or lacrimal duct surgery, use of contact lenses in the last 2 weeks, long-term history of ocular medication, among others |
| **Follow up** | Patients were followed for 3 months and were examined before treatment, at 1 month, and at 3 months after the last treatment. |
| **Intervention** | IPL+ Meibomian Gland Expression  3 sessions, 3 weeks apart |
| **Participants, n** | 35 patients |
| **Drop-outs, n (%)** | 1 (2.86%) |
| **Comparison** | Meibomian Gland Expression  The study also had a group that received IPL only (not reported here). |
| **Participants, n** | 32 patients |
| **Drop-outs, n (%)** | 2 (6.25%) |
| **Primary outcomes** | **Dry eye symptoms** (OSDI, min= 0; max= 100; higher= worse); mean (SD):  Baseline  I (n= 35)= 28.19 (16.77)  C (n= 32)= 23.38 (17.39)  Follow up at 3 months after the last treatment  I (n=)= 15.93 (14.60)  C (n= )= 15.83 (13.37)  Group difference; mean(95%CI)= NA |
| **Reported adverse events** | Mild pain and burning sensation during the IPL therapy, but no sustained skin injury occurred after the treatment. Overall, no complications were observed. |
| **Comments** |  |
| **Risk of bias** | Some concerns |

| **Author** | **Ally L. Xue** |
| --- | --- |
| **Year** | 2020 |
| **Country** | New Zealand |
| **Study design** | RCT |
| **Setting** | Department of Ophthalmology, New Zealand National Eye Centre, The University of Auckland, New Zealand |
| **Recruitment** | Participants were recruited prospectively from the community |
| **Population** | **Gender n (%): 58 females (66.7%), 29 males (33.3%)**  **Mean age (SD)**: 53 (16 years)  **Diagnosis:** Meibomian gland dysfunction  **Severity of symptoms**: not specified  **Duration of symptoms**: not specified |
| **Inclusion criteria** | Age 18 years or older with symptoms of dry eye disease (McMonnies dry eye questionnaire score ≥10 and/or Ocular Surface Disease Index score ≥13); Clinically significant signs of MGD(eyelid margin or mucocutaneous junction abnormalities, meibomian gland orifice capping, and/or decreased expressed meibum quality); No contact lens wear or use of systemic medications known to affect the eye; Non-pregnant; No history of major systemic, dermatologic, or ocular conditions; No ocular surgery or dermatologic treatments in the previous three months; No implants, tattoos, semi-permanent makeup, or pigmented lesions in the treatment area; No contraindications to IPL therapy, including the use of photosensitive medications.  **Exclusion**: Not specified in the text |
| **Follow up** | Assessments were conducted on days 0, 15, 45, 75, and four weeks after treatment course completion on day 105. |
| **Intervention** | Intense Pulsed Light (IPL) therapy with four or five homogeneously sequenced light pulses on days 0, 15, 45, and 75.E-Eye Intense Regulated Pulsed Light, E-Swin, France |
| **Participants, n** | 4 flashes group (n= 28)  5 flashes group (n=29) |
| **Drop-outs, n (%)** | NONE |
| **Comparison** | placebo treatment |
| **Participants, n** | n= 30 |
| **Drop-outs, n (%)** | none |
| **Primary outcomes** | **Dry eye symptoms** (OSDI, min= 0; max= 100; higher= worse); mean (SD):  Baseline  I with 4 flashes (n= 28)= 28 (16)  I with 5 flashes (n= 29)= 28 (20)  C (n= 30)= 34 (16)  Follow up four weeks after treatment course completion on day 105  I with 4 flashes (n= 28)= 22 (18)  I with 5 flashes (n= 29)= 21 (17)  C (n= 30)= 31(22)  Group difference; mean(95%CI)= NA for the comparison of interest |
| **Reported adverse events** | No adverse effects |
| **Comments** |  |
| **Risk of bias** | Some concerns |

| **Author** | **Bei Rong (a)** |
| --- | --- |
| **Year** | 2018 |
| **Country** | China |
| **Study design** | Prospective, randomized, double-masked, controlled study |
| **Setting** | Departments of Ophthalmology and Dermatology, Peking University First Hospital, Beijing, China |
| **Recruitment** | Consecutive MGD patients treated at the Ophthalmology Department of Peking University First Hospital between March and July 2016. |
| **Population** | **Gender n (%):**12 men (27%) and 32 women (73%)  **Mean age (SD)**: 46.3 (16.9)  **Diagnosis: Meibomian gland dysfunction (MGD)**  **Severity of symptoms**: Chronic, diffuse abnormality of the meibomian gland  **Duration of symptoms**: Not stated |
| **Inclusion criteria** | Age above 18 years; Obstruction of MG orifices observed under slit lamp examination; Meibomian gland yielding secretion score (MGYSS) of lower eyelid of no more than 12; Standard Patient Evaluation of Eye Dryness (SPEED) questionnaire score of at least 6 in both eyes; Fitzpatrick skin types 1–4 according to sun sensitivity and appearance of the skin  **Exclusion**: Any intraocular inflammation, ocular surgery, or ocular trauma in the past 6 months; Ocular infection or allergy; Any eyelid structural abnormality; Any systemic diseases that may lead to dry eye disease; Tanning in the past 4 weeks; Skin cancer or pigmented lesion in the treatment zone; Pregnancy or lactation. |
| **Follow up** | Not stated, but the study involved three treatment sessions at 4-week intervals. |
| **Intervention** | Intense Pulsed Light (IPL) Meibomian Gland Expression, and artificial tears  IPL was applied directly on the eyelids using the M22 IPL system with optimal pulse technology, set to 14–16 J/cm2, and a 560-nm filter. The eye was protected with a Jaeger lid plate during treatment. |
| **Participants, n** | the study involved 44 patients with one eye randomly selected for IPL treatment and the other serving as a control. |
| **Drop-outs, n (%)** | Two patients quit the study for reasons not related to the study and were not included in the analysis. (4.55%) |
| **Comparison** | sham IPL treatment, Meibomian Gland Expression, and artificial tears  three times a  day, in both eyes. |
| **Participants, n** | the study involved 44 patients with one eye randomly selected for IPL treatment and the other serving as a control. |
| **Drop-outs, n (%)** | Two patients quit the study for reasons not related to the study and were not included in the analysis (4.55%) |
| **Primary outcomes** | **Dry eye symptoms** (SPEED, min= 0; max= 28; higher= worse); mean (SD):  Baseline  I (n=42 eyes)= 17.4 (5.55)  C (n= 42 eyes)=17.4 (5.55)  Follow up, mean difference from baseline (SD; not clear if SD is that of the difference or of the mean):  I (n= 42 eyes)= -10.1 (6.7)  C (n= 42 eyes)= -10.0 (6.7)  Group difference; mean(95%CI)= NA; p= 0.510 |
| **Reported adverse events** | Mild pain and burning during IPL treatment in five patients, mild redness of the eyelids in their study eyes, and one patient suffered partial eyelash loss. No irreversible eyelid skin injury occurred, and no intraocular inflammation, iris transillumination defects, or ocular surface or fundus injuries were observed. |
| **Comments** | Wrong unit of analysis: Intervention and control data is from the same patients because they had one intervention eye and one control eye. Same cohort as in the reference Rong (b) 2018. |
| **Risk of bias** | Some concerns |

| **Author** | **Bei Rong (b)** |
| --- | --- |
| **Year** | 2018 |
| **Country** | China |
| **Study design** | Prospective, randomized, double-masked, controlled study |
| **Setting** | Department of Ophthalmology, Peking University First Hospital, Beijing, China |
| **Recruitment** | Patients were recruited from the Department of Ophthalmology of Peking University First Hospital between January 2016 and April 2017. |
| **Population** | **Gender n (%):**10 men (35.7%), 18 women (64.3%)  **Mean age (SD)**: 42.17 years (17.62)  **Diagnosis: Meibomian Gland Dysfunction (MGD)**  **Severity of symptoms**: Not mentioned  **Duration of symptoms**: Not mentioned |
| **Inclusion criteria** | Age above 18 years; Standard Patient Evaluation of Eye Dryness (SPEED) questionnaire score of at least 6 for both eyes; Meibomian gland yielding secretion score (MGYSS) of no more than 12 for the lower eyelid; Fitzpatrick skin type 1–4;  **Exclusion**: Intraocular inflammation, ocular surgery, or ocular trauma in the past 6 months; Ocular infection or allergy; Any eyelid structural abnormality; Any systemic diseases that may lead to dry eye disease; Tanning in the 4 weeks before enrollment; Skin cancer or pigmented lesion in the treatment zone; Pregnancy or lactation |
| **Follow up** | 9 months |
| **Intervention** | Intense Pulsed Light (IPL) Meibomian Gland Expression, and artificial tears  IPL was applied directly on the eyelids using the M22 IPL system with optimal pulse technology, set to 14–16 J/cm2, and a 560-nm filter. The eye was protected with a Jaeger lid plate during treatment. |
| **Participants, n** | 44 |
| **Drop-outs, n (%)** | 16 (36.36%) -28 patients completed the entire 9-month follow-up assessment and were included in the analysis. |
| **Comparison** | sham IPL treatment, Meibomian Gland Expression, and artificial tears |
| **Participants, n** | 44 |
| **Drop-outs, n (%)** | 16 (36.36%) |
| **Primary outcomes** | **Dry eye symptoms** (SPEED, min= 0; max= 28; higher= worse); mean (SD):  Baseline  I (n= 42 eyes)= 16.82 (5.5)  C (n= 42 eyes)=16.82(5.5)  Follow up  I (n= 28 eyes)= -10.04  C (n= 28 eyes)= -9.71  Group difference; mean(95%CI): NA |
| **Reported adverse events** | No serious adverse ocular and dermal effects were detected during the study. |
| **Comments** | 28 patients completed the entire 9-month follow-up assessment and were included in the analysis.- long term continuation from RONG short  Wrong unit of analysis: Intervention and control data is from the same patients because they had one intervention eye and one control eye. Same cohort as in the reference Rong (a) 2018. |
| **Risk of bias** | High |

| **Author** | **Piyacomn** |
| --- | --- |
| **Year** | 2020 |
| **Country** | Thailand |
| **Study design** | Randomized, double-masked, sham-controlled clinical trial |
| **Setting** | Department of Ophthalmology, Faculty of Medicine, Chulalongkorn University, and King Chulalongkorn Memorial Hospital, Bangkok, Thailand |
| **Recruitment** | Consecutively enrolled from the eye clinic, Department of Ophthalmology, King Chulalongkorn Memorial Hospital, Thailand, from August 2018 to March 2019 |
| **Population** | **Gender n (%):** Not stated  **Mean age (SD)**: Not stated  **Diagnosis:** Meibomian gland dysfunction (MGD)  **Severity of symptoms**: Stages 1–4 of MGD  **Duration of symptoms**: Not state |
| **Inclusion criteria** | Age between 18 and 80 years; Fitzpatrick skin types 1 to 5; Able and willing to comply with the treatment/follow-up schedule and requirements; Presence of meibomian glands on each lower eyelid’s meibography; Diagnosed at any stage of MGD in both eyes, according to the International Workshop on MGD  **Exclusion**: Contact lens wearers within the past 1 month and throughout the study; Use of any antiglaucoma eye drops within the past 3 months and throughout the study period; Recent ocular or eyelid surgery, neuroparalysis in the planned treatment area, and subjects who have undergone refractive surgery within the past 6 months; IPL treatment and single-dose vectored thermal pulsation treatment or any equivalent treatments within the past 12 months; Current use of punctal plugs, presence of precancerous lesions, skin cancer, or pigmented lesions in the planned treatment area, uncontrolled infections or uncontrolled immunosuppressive diseases, diseases in the planned treatment area that could be stimulated by light, use of photosensitive medications and/or herbs such as isotretinoin or tetracycline, pregnancy and lactation, radiation therapy to the head or neck within the past year or planned radiation therapy throughout the study period, treatment with a chemotherapeutic agent within the past 8 weeks or planned chemotherapy throughout the study period, and declared legally blind in 1 eye |
| **Follow up** | 3 months (also on day 0, day 15 and 6 months) |
| **Intervention** | Intense pulsed light (IPL) + artificial tears 4 times daily  IPL ttreatment was performed on days 0, 15, and 45. using the E.Eye (E-SWIN, Paris, France) device, applied below the inferior lid margin divided into 5 areas according to the company's recommendations. |
| **Participants, n** | 57 |
| **Drop-outs, n (%)** | 2 (3.51%) |
| **Comparison** | sham IPL on days 0, 15, and 45 + artificial tears 4 times daily |
| **Participants, n** | 57 |
| **Drop-outs, n (%)** | 7 (12.28%) |
| **Primary outcomes** | **Dry eye symptoms** (OSDI, min= 0; max= 100; higher= worse); mean (SD):  Baseline  I (n= 57) = 38.76 (21.11)  C (n= 53) = 36.02 (21.28)  Follow up at month 3  I (n=57) = 25.04 (16.97)  C (n= 53) = 29.12 (16.89)  Group difference; mean(95%CI)= -3.91 (-11.11 to 3.28) |
| **Reported adverse events** | No adverse events occurred after IPL treatment |
| **Comments** |  |
| **Risk of bias** | Low |

| **Author** | **Toyos** |
| --- | --- |
| **Year** | 2022 |
| **Country** | United States |
| **Study design** | Prospective, interventional, multi-site, parallel-group, two-arms, randomized, active-controlled with a 1:1 allocation ratio. |
| **Setting** | The study was conducted at three clinics in the USA: Dell Laser Consultants in Austin, Texas; Toyos Clinic in Nashville, Tennessee; Eye Institute of West Florida in Largo, Florida. |
| **Recruitment** | Patients were recruited between January 2018 and May 2019. |
| **Population** | **Gender n (%):** In the control arm, there were 33 women (76.7%) and 10 men (23.3%). In the study arm, there were 22 women (56.4%) and 17 men (43.6%)  **Mean age [95% confidence interval]**: control= 56.8 [52.9, 60.7]; Intervention= 54.3 years [49.8, 58.7]  **Diagnosis:** Dry eye disease due to MGD (Meibomian Gland Dysfunction)  **Severity of symptoms**: Moderate to severe symptoms of dry eye.  **Duration of symptoms**: Not mentioned |
| **Inclusion criteria** | Adults aged 22 to 85 years with signs and symptoms of dry eye disease due to MGD; Tear break-up time (TBUT) ≤ 7 seconds in the study eye; Meibomian gland secretion (MGS) ≥ 12 in the study eye (evaluated based on Lane et al.'s scoring system); At least 5 non-atrophied meibomian glands in the lower eyelid of the study eye; Ocular Surface Disease Index (OSDI) questionnaire score ≥ 23 (indicating moderate to severe symptoms of dry eye).  **Exclusion**: Fitzpatrick skin type V or VI; Use of prescription eye drops within 7 days prior to recruitment (excluding artificial tears or glaucoma drops); Facial IPL treatment within the past 12 months; Any thermal treatment of the eyelids or meibomian gland expression within the past 6 months; Ocular surface and eyelid abnormalities, any systemic condition that may cause dry eye; Use of photosensitive drugs within the past 3 months; Pre-cancerous lesions, skin cancer, or pigmented lesions within the treatment area; Overexposure to the sun within the past 1 month; Ocular infections within the past 6 months; Uncontrolled infections or immunosuppressive diseases; Unwillingness or inability to abstain from the use of medications known to cause dryness. |
| **Follow up** | 4 weeks after the fourth treatment session |
| **Intervention** | IPL treatment followed by meibomian gland expression. Each patient underwent four treatment sessions, two weeks apart. IPL treatment was administered using a Lumenis M22 system with specific parameters. Treatment areas included the malar region and the peri-ocular area |
| **Participants, n** | 45 patients |
| **Drop-outs, n (%)** | 6 (13.33%) |
| **Comparison** | sham IPL followed by meibomian gland expression |
| **Participants, n** | 43 patients |
| **Drop-outs, n (%)** | NONE |
| **Primary outcomes** | **Dry eye symptoms** (OSDI, min= 0; max= 100; higher= worse); mean [95%CI]:  Baseline  I (n= 39 )= 53.8 [47.1, 60.5]  C (n= 43) = 60.2 [54.6, 65.9]  Follow up  I (n= 39) = 27.9 [21.5, 34.3]  C (n= 43) = 34.3 [27.5, 41.1]  Group difference; mean(95%CI)= NA, p= 0.9984 |
| **Reported adverse events** | not mentioned |
| **Comments** |  |
| **Risk of bias** | High |

| **Author** | **X. Yan et al.** |
| --- | --- |
| **Year** | 2021 |
| **Country** | China |
| **Study design** | Randomized Controlled Trial (RCT) |
| **Setting** | Multi-center study conducted in four sites in China |
| **Recruitment** | Subjects were recruited between November 2017 and April 2018 |
| **Population** | **Gender n (%):** women=92 (77%); men= 28 (23%)  **Mean age (SD)**: I= 42.46 years (14.2); C= 41.86 years (14.1)  **Diagnosis:** meibomian gland dysfunction (MGD)  **Severity of symptoms**: not mentioned  **Duration of symptoms**: not mentioned |
| **Inclusion criteria** | Male or female subjects over the age of 18; Fitzpatrick skin types I-IV; Symptoms of Dry eye disease (SPEED score of at least six points); Bilateral evidence of meibomian gland obstruction; Bilateral TBUT shorter than 10 sec; Bilateral evidence of corneal punctate staining or corneal lesions on CFS examination.  **Exclusion**: Contraindications to IPL; Recent or current dry eye management; Obvious scar or severe keratinization of the lid margin; Pregnant/lactating women; Subjects with ocular surface diseases (OSDs) |
| **Follow up** | 12 weeks after the baseline |
| **Intervention** | Intense pulsed light (IPL) therapy (with a M22 OPT system) followed by manual expression of the meibomian glands 3 times at the clinic, at 3-week intervals + artificial tears (Systane, Alcon, 5 mL) 3 times daily. |
| **Participants, n** | 60 |
| **Drop-outs, n (%)** | 1 (1.67%) |
| **Comparison** | Warm compress therapy followed by manual expression of the meibomian glands 3 times at the clinic, at 3-week intervals + artificial tears (Systane, Alcon, 5 mL) 3 times daily |
| **Participants, n** | 60 |
| **Drop-outs, n (%)** | 5 (8.33%) |
| **Primary outcomes** | **Dry eye symptoms** (SPEED, min= 0; max= 28; higher= worse); mean (SD):  Baseline  I (n= 60)= 15.2 (4.7)  C (n= 60)= 14.1 (4.9)  Follow up  I (n= 59)= 9.2 (4.8)  C (n= 55)= 10.7 (4.7)  Group difference; mean(95%CI)= NA; p=0.08 |
| **Reported adverse events** | No adverse events related to the device or the procedure.  In the control group, one patient developed lower eyelid edema during the study and had to stop participation in the study. |
| **Comments** |  |
| **Risk of bias** | Some concerns |

| **Author** | **Song** |
| --- | --- |
| **Year** | 2022 |
| **Country** | China (with participation from the United Kingdom) |
| **Study design** | Randomized, single-masked, sham-controlled study |
| **Setting** | He Eye Specialist Hospital, Shenyang, China |
| **Recruitment** | Consecutive subjects recruited from He Eye Specialist Hospital, Shenyang outpatient department between January 2019 to January 2020. |
| **Population** | **Gender n (%):** not specified  **Mean age (SD)**: IPL group: 28.16 (3.59) years; Sham group: 28.07 (3.71) years  **Diagnosis:** Dry eye disease (DED) due to meibomian gland dysfunction (MGD)  **Severity of symptoms**: Not stated  **Duration of symptoms**: Not stated |
| **Inclusion criteria** | Age ≥ 18 years; Fitzpatrick skin types 1 to 4; Willing to comply with treatment/follow-up schedule; Diagnosis of Dry eye disease based on ocular symptoms, NITBUT ≤5 sec, fluorescein staining score ≥1, visualization of meibomian glands, bilateral diagnosis of any stage of MGD.  **Exclusion**: Excluded: Fitzpatrick skin type 5, eyelid structural abnormality, intraocular inflammation, recent ocular surgery or trauma, ocular infection or allergy, certain systemic diseases or medications, pterygium, corneal neovascularization, glaucoma, rheumatic immune systemic diseases, history of herpes zoster infection, skin cancer, pregnancy or breastfeeding, fluorescein allergy, contact lens wear |
| **Follow up** | 3 months |
| **Intervention** | IPL treatment + artificial tears  IPL with 12 homogeneously spaced pulsed light to both eyes (with a M22 system), utilizing the Toyos protocol, administered at 3-week intervals (day-0, day-21, day-42) |
| **Participants, n** | 53 |
| **Drop-outs, n (%)** | 8 (15.09%) |
| **Comparison** | Sham treatment + artificial tears |
| **Participants, n** | 53 |
| **Drop-outs, n (%)** | 12 |
| **Primary outcomes** | **Dry eye symptoms** (OSDI, min= 0; max= 100; higher= worse); mean (SD):  Baseline  I (n= 45)= 35.40 (9.39)  C (n= 41)= 34.38 (9.57)  Follow up  I (n= 45)= 19.02 (6.7)  C (n= 41)= 35.13 (9.41)  Group difference; mean (95%CI)= NA; F =169.557; p< 0.001-15.93 |
| **Reported adverse events** | 0 significant changes or adverse effects observed |
| **Comments** |  |
| **Risk of bias** | Some concerns |

| **Author** | **Li** |
| --- | --- |
| **Year** | 2023 |
| **Country** | China |
| **Study design** | Randomized controlled study |
| **Setting** | Dry Eye Clinic, Department of Ophthalmology, He Eye Specialist Hospital, Shenyang, China |
| **Recruitment** | Participants diagnosed with dry eye were recruited from January to October 2021 at the Dry Eye Clinic. |
| **Population** | **Gender n (%):**IPL= 28 Females (56%); CONTROL= 27 Females (54%)  **Mean age (SD)**: IPL: 29.88 (4.68) years, Control: 28.52 (3.77) years  **Diagnosis:** Dry eye disease (DED), specifically evaporative dry eye (EDE)  **Severity of symptoms**: mild to severe  **Duration of symptoms**: not mentioned |
| **Inclusion criteria** | Age <18 years; Fitzpatrick skin types I–IV; Capable and willing to comply with treatment and follow-up obligations; Determination of severe Dry eye disease based on: Ocular Surface Disease Index (OSDI; a score of 33 indicates severe Dry eye disease), Noninvasive tear film breakup time (NITBUT) of <5 sec, Corneoconjunctival staining (CS) score of <3 points according to the Asian Dry Eye Consensus.  **Exclusion**: Existing ocular trauma, infectious diseases, and recent surgical history; Skin defects, pigmentation, moles, scars in the treatment area, and skin cancer; Autoimmune diseases and skin allergies; Pregnancy or breastfeeding; Fitzpatrick skin types V or VI. |
| **Follow up** | 84 days (D0, D21, D42, D84) |
| **Intervention** | IPL therapy. IPL sessions were administered on D0, D21, and D42 with a M22 system (Lumenis, Yokneam, Israel) with a xenon lamp emitting light with a wavelength ranging from 515 to 1200 nm.  (a second group, not used in this review, received IPL with heated eye mask (HEM)). |
| **Participants, n** | 50 eyes |
| **Drop-outs, n (%)** |  |
| **Comparison** | control group (no treatment) |
| **Participants, n** | 50 eyes |
| **Drop-outs, n (%)** |  |
| **Primary outcomes** | **Dry eye symptoms** (OSDI, min= 0; max= 100; higher= worse); mean (SD):  Baseline  I (n= 50)= 32.30  C (n= 50)= 33.42  Follow up at day 42  I (n= 50)= 27.16 (3.90)  C (n= 50)= 35.04 (8.84)  Follow up at day 84  I (n= 50)= 22.56 (4.60)  C (n= 50)= 36.08 (7.83)  Group difference; mean(95%CI)= NA |
| **Reported adverse events** | Not mentioned |
| **Comments** |  |
| **Risk of bias** | Some concerns |

| **Author** | **Craig** |
| --- | --- |
| **Year** | 2015 |
| **Country** | New Zealand |
| **Study design** | Prospective, double-masked, placebo-controlled, paired-eye study |
| **Setting** | Ocular Surface Laboratory, Department of Ophthalmology, New Zealand National Eye Centre, University of Auckland, Auckland, New Zealand |
| **Recruitment** | Not mentioned |
| **Population** | **Gender n (%):** 20 female participants (no mention of male participants)  **Mean age (SD)**: 45 (15) years  **Diagnosis:** Meibomian Gland Dysfunction (MGD)  **Severity of symptoms**: Mild to moderate  **Duration of symptoms**: Not mentioned |
| **Inclusion criteria** | Participants with mild to moderate clinical signs of Meibomian Gland Dysfunction (MGD).  **Exclusion**: Individuals for whom light therapy was contraindicated; Those who had received clinical skin treatments within the prior 2 months; Individuals with implants beneath the treatment area; Those with tattoos, semipermanent makeup, or pigmented lesions in the treatment area;Contact lens wearers within 48 hours of commencing the study or during the study. |
| **Follow up** | 45 days |
| **Intervention** | Intense pulsed light (IPL) applied to the periocular area, during three separate treatment sessions on Day (D) 1, D15, and D45, using a third-generation IPL device designed specifically for periocular application with multiple homogenously sculpted light pulses (E>Eye; E-SWIN, Paris, France) |
| **Participants, n** | 28 participants |
| **Drop-outs, n (%)** | none |
| **Comparison** | Comparison between treated eyes and control eyes (sham) |
| **Participants, n** | 28 participants |
| **Drop-outs, n (%)** | none |
| **Primary outcomes** | **Dry eye symptoms** (SPEED, min= 0; max= 28; higher= worse); mean (SD):  **At follow-up on day 45**  I (n= 28 eyes)= 8.5 (NA)  C (n= 28 eyes)= 8.5 (NA)  Group difference; mean(95%CI)= NA; “no significant differences between the treated and control eye SPEED scores at any visit.” |
| **Reported adverse events** | Not mentioned |
| **Comments** | Excluded from meta-analysis for wrong unit of analysis |
| **Risk of bias** | Some concerns |

| **Author** | **Arita** |
| --- | --- |
| **Year** | 2019 |
| **Country** | Japan |
| **Study design** | Prospective, randomized controlled study |
| **Setting** | Conducted at Itoh Clinic, Saitama, Japan |
| **Recruitment** | Patients with refractory MGD attending Itoh Clinic were enrolled |
| **Population** | **Gender n (%):** I= female 9 (41%) male 13 (59%); C= female 8 (40%) male 12 (60%)  **Mean age (SD)**: 61.0 (18.0)  **Diagnosis:** Refractory meibomian gland dysfunction (MGD)  **Severity of symptoms**: Refractory MGD, failed to respond over a period of at least 2 years to at least three types of conventional therapy.  **Duration of symptoms**: Not stated |
| **Inclusion criteria** | Age of at least 20 years; Diagnosis of MGD according to Japanese MGD diagnostic criteria; Fitzpatrick skin type of 1–4; Refractory MGD as defined by the failure to respond over a period of at least 2 years to at least three types of conventional therapy.  **Exclusion**: Not stated |
| **Follow up** | 24 weeks after treatment onset (data also available for 32 weeks) |
| **Intervention** | Combination of intense pulsed light (IPL) and meibomian gland expression (MGX). IPL therapy was administered as a series of eight treatment sessions at 3-week intervals. using the M22 IPL machine (Lumenis, Yokneam, Israel) with a range of 11–14 J/cm². MGX was performed using an Arita Meibomian Gland Compressor (Katena, Denville, NJ). |
| **Participants, n** | 22 |
| **Drop-outs, n (%)** | None |
| **Comparison** | Meibomian gland expression alone |
| **Participants, n** | 23 patients |
| **Drop-outs, n (%)** | 3 (13.04%) |
| **Primary outcomes** | **Dry eye symptoms** (SPEED, min= 0; max= 28; higher= worse); mean (SD):  I Baseline  I (n= 22)= 14.7 (3.4)  C (n= 20)= 12.7 (4.8)  Follow up  (n= 22)= 5,9 (6.0)  C (n= 20)= 9.1(3.8)  Group difference; mean(95%CI)= NA; p= 0.24 |
| **Reported adverse events** | No adverse events were reported |
| **Comments** |  |
| **Risk of bias** | Some concerns |

| **Author** | **Zarei-Ghanavati** |
| --- | --- |
| **Year** | 2022 |
| **Country** | Iran |
| **Study design** | Randomized controlled trial |
| **Setting** | University hospital tertiary referral center |
| **Recruitment** | Participants were enrolled based on clinical diagnosis and met eligibility criteria. |
| **Population** | **Gender n (%):** Males: 31 (31%), Females: 69 (69%)  **Mean age (SD)**: 44 (15)  **Diagnosis:** Meibomian gland dysfunction (MGD)  **Severity of symptoms**: Mild, moderate, and severe MGD  **Duration of symptoms**: Not mentioned |
| **Inclusion criteria** | Symptomatic patients with a clinical diagnosis of mild, moderate, and severe Meibomian Gland Dysfunction (MGD); Patients who did not receive any dry eye treatment for at least one month; Participants aged 18 or older; Participants with clinically significant signs of MGD; Participants with signs and symptoms of dry eye disease, including reporting symptoms of ocular surface discomfort and an Ocular Surface Disease Index (OSDI) score ≥ 13; Participants with a fluorescein tear break-up time (FTBUT) < 10 seconds.  **Exclusion**: Patients with active ocular infection or disease other than dry eye; Patients with a history of ophthalmic surgery; Patients who used contact lenses within 3 months of or during the study; Patients with any systemic disease including diabetes and autoimmune disease; Patients using systemic, photosensitizing, or ocular medications except unpreserved lubricants one month before and during the study; Patients with contraindications to IPL therapy, including skin tattoos or cosmetic procedures in the treatment area, lactation, pregnancy, and dark skin type (Fitzpatrick skin type VI). |
| **Follow up** | Day 75 (data also available for Day 15 and 45) |
| **Intervention** | Three sessions on day 0, 15 and 45, of in-office IPL therapy using the E-Eye IPL machine, with five sequential, overlapping IPL flashes applied to the inferior and temporal preocular area. IPL therapy was combined with conventional home-based therapy |
| **Participants, n** | 50 |
| **Drop-outs, n (%)** | none |
| **Comparison** | Conventional home care |
| **Participants, n** | 50 |
| **Drop-outs, n (%)** | 3 (6%) |
| **Primary outcomes** | **Dry eye symptoms** (OSDI, min= 0; max= 100; higher= worse); mean (SD):  Baseline  I (n= 50)= 41.3  C (n= 47)=43.7  Follow up at day 75  I (n= 50)= 14.8 (11.6)  C (n= 47)= 20.6 (14.0)  Group difference; mean(95%CI)= NA; p(ANOVA treatment x time)=0.071 |
| **Reported adverse events** | No significant adverse events were reported |
| **Comments** |  |
| **Risk of bias** | Some concerns |

| **Author** | **Chen** |
| --- | --- |
| **Year** | 2023 |
| **Country** | China |
| **Study design** | Prospective, randomized, controlled trial |
| **Setting** | He Eye Specialist Hospital, Shenyang, China |
| **Recruitment** | Participants were recruited at the Department of Ophthalmology, He Eye Specialist Hospital, Shenyang, China. |
| **Population** | **Gender n (%):** I= 11 (52%) female; C= 9 (50%) female  **Mean age (SD)**: I= 32.23 (7.29) years ; C=32.26 (6.67) years  **Diagnosis:** Participants diagnosed with dry eye disease (DED)  **Severity of symptoms**: severe evaporative dry eye (EDE)  **Duration of symptoms**: Not mentioned |
| **Inclusion criteria** | Age ≥ 18 years; Fitzpatrick skin types I–IV; Able and willing to comply with treatment and follow-up schedule; Bilateral diagnosis at any stages of meibomian gland dysfunction (MGD); Bilateral signs and symptoms of dry eye disease  **Exclusion**: Existing ocular trauma, infectious diseases, recent surgical history; Skin defects, pigmentation, moles, scars in the treatment area, skin cancer; Autoimmune diseases, skin allergies; Pregnancy or lactation; Fitzpatrick skin type IV and V; Patients with corneal refractive surgery such as laser-assisted in situ keratomileusis (LASIK). |
| **Follow up** | 28 days |
| **Intervention** | Intense pulsed light (IPL) therapy for 2 sessions 2 weeks apart (using a M22 IPL system by Lumenis Ltd.) |
| **Participants, n** | 22 participants (44 eyes) |
| **Drop-outs, n (%)** | none |
| **Comparison** | IPL sham therapy |
| **Participants, n** | 22 participants (44 eyes) |
| **Drop-outs, n (%)** | none |
| **Primary outcomes** | **Dry eye symptoms** (OSDI, min= 0; max= 100; higher= worse); mean (SD):  Baseline  I (n=)= 42.97 (8.78)  C (n= )= 42.36 (7.10)  Follow up  I (n=)= 24.77 (4.68)  C (n= )= 42.61 (7.49)  Group difference; mean(95%CI)= NA for the comparison of interest |
| **Reported adverse events** | No systemic adverse events were observed during the study, although rare cases of eye irritation, conjunctival hyperemia, eye pain, and sensitivity or fragility of the skin around the eye were reported. |
| **Comments** | Not clear if the means and standard deviations reported with participant or eye as unit of analysis. Because SD are similar to the studies reporting participants as unit of analysis, data was treated as such. |
| **Risk of bias** | Some concerns |
