# Supplementary figures and images for: Effectiveness and safety of intense pulsed light therapy for dry eye symptoms due to meibomian gland dysfunction—A systematic review and meta‐analysis
Source: Acta Ophthalmol. 2024 Nov 29;103(4):371–9. doi: 10.1111/aos.16802 (PMC12069959; doi:10.1111/aos.16802)

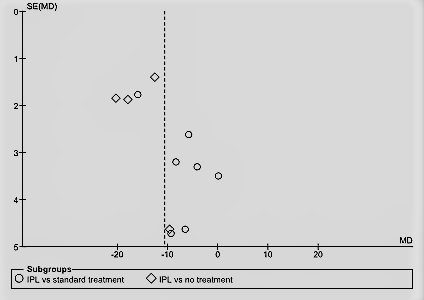


**Supporting Information S4: Funnel plot**

Supplement: Supplementary file 4 — Data S4. [file AOS-103-371-s004.docx]
